# The impact of collaborative care on perceptions of treatment across racial and ethnic groups

**DOI:** 10.3389/frhs.2026.1813110

**Published:** 2026-04-30

**Authors:** Maya Rabinowitz, Rebecca L. Weir, Sapna J. Mendon-Plasek, Colleen M. McCullough, Lauren Kelly, Beth Ann Griffin, Lia Pak, P'trice R. Jones, Katherine E. Watkins

**Affiliations:** 1 Department of Psychology, Yale University, New Haven, CT, United States; 2 RAND, Arlington, VA, United States; 3 RAND, Boston, MA, United States; 4 RAND, Santa Monica, CA, United States

**Keywords:** clinician relationships, CoCM, ethnicity, OUD, race

## Abstract

**Introduction:**

This study examined the impact of the Collaborative Care Model (CoCM) on patients' subjective perceptions of clinician communication, treatment timeliness, and overall treatment quality, with a particular focus on whether these effects differ across racial and ethnic groups.

**Methods:**

797 adult participants with opioid use disorder (OUD) co-occurring with major depression and/or PTSD were recruited for the study from 18 rural and urban primary care clinics in New Mexico and California. Following enrollment, patients were randomized to either CoCM or enhanced usual care (EUC). Using linear models, we examined the relationship between treatment condition and patient perceptions of clinician communication, treatment timeliness, and overall treatment quality among a subset of 503 patients with follow-up measures at 6-months. We also examined whether this relationship was moderated by race and ethnicity.

**Results:**

We found no evidence for an effect of CoCM compared to EUC on patients' perceptions of clinician communication, treatment timeliness, or overall treatment quality, and no evidence of moderation by patient race or ethnicity.

**Conclusion:**

The present results conflict with prior literature suggesting that collaborative care improves patient perceptions of clinical experiences across diverse patient samples. These conflicting findings highlight the need for localized research that considers specific patient populations and social contexts when examining the impacts of collaborative care on subjective patient perceptions.

## Introduction

The Collaborative Care Model (CoCM) is a team-based healthcare model that integrates behavioral health services into primary care settings (1). The care team typically includes a primary care provider, a care manager who advocates for the patient and coordinates the team, and a psychiatric consultant. In health systems using CoCM, these providers work together to facilitate comprehensive and coordinated treatment (2). Across multiple behavioral health conditions [e.g., depression, anxiety, and Post-Traumatic Stress Disorder (PTSD)], CoCM is associated with stronger treatment adherence (3, 4), reduced treatment cost (5) and improved physical and behavioral health outcomes (6–9), compared to usual care. Notably, CoCM has also been shown to improve health outcomes for racially and ethnically minoritized patients (10, 11), and in some cases, reduce racial disparities in health outcomes (12, 13).

Beyond these clinical outcome metrics, an expanding body of research has begun to unpack the impact of CoCM on patients' *subjective* perceptions of treatment (3, 8, 14). This work aligns with a broader literature recognizing patient perceptions as a fundamental indicator of care quality (15–17), and emphasizing the importance of patient satisfaction and clinician relationships as core components of high-quality care (18–20). Investigations on the impacts of CoCM on subjective patient experiences have largely concluded that CoCM leads to overall greater patient satisfaction (3, 8, 14) and specifically, improved clinician relationships (3, 21), compared to usual care. Two key components of these clinician relationships include *clinician communication*—the language that providers use when interacting with patients (22)—and *treatment timeliness*—the recognized urgency and prompt attention given to patients by providers (23, 24). Research has found CoCM to be associated with both improved perceptions of clinician communication (3, 21) and treatment timeliness (25), which themselves predict greater treatment adherence, patient retention, and overall health outcomes (23, 24, 26).

A growing body of literature suggests that perceptions of care—and specifically, perceptions of clinician relationships, including clinician communication and treatment timeliness—can vary significantly between patients of different races and ethnicities (2, 27–31). Specifically, research suggests that compared to White patients, Black and Latine patients generally report poorer quality clinician communication (31–33). This disparity stems in part from the racialized disrespect that many non-White patients perceive from providers (29), and from a broader lack of culturally-competent communication that centers marginalized patients' cultural needs, experiences, and lived reality (34–39). Black and Latine patients also report poorer treatment timeliness—longer wait times, less attention, and generally less speedy treatment compared to White patients (38, 40, 41). Experiencing such racial bias in healthcare settings can lead minoritized patients to report distrust in the healthcare system (42, 43), and is associated with reduced treatment adherence and retention, and ultimately worse health outcomes (44–48).

Importantly, the experience of bias in healthcare settings is often further exacerbated among racially and ethnically minoritized patients whose health conditions carry additional stigma (49). The consequences of stigma for illnesses such as mental health and substance use disorders may be worse for racially and/or ethnically minoritized patients compared to racial and/or ethnic majorities (49, 50). In these contexts, assumptions and stereotypes about both racial identity and health disorder may further intensify negative clinician relationships and clinical experiences (50, 51).

Given this social context, explorations of the impact of CoCM on perceived clinician relationships must consider how the effects are patterned by race and ethnicity, particularly among patients whose identities intersect with stigmatized health conditions. A small body of research has found collaborative care to improve treatment satisfaction among racially or ethnically minoritized patients (52, 53). However, to our knowledge, no research has explicitly tested whether race or ethnicity moderates the effect of CoCM on subjective perceptions of clinician relationships, especially among patients with stigmatized health conditions such as co-occurring substance use and mental health disorders.

It is possible that CoCM reduces often-observed racial and ethnic disparities in clinician communication and treatment timeliness (31–33, 41). Since one crucial part of CoCM is the presence of a care manager—often a nurse, social worker, or community health worker who advocates on behalf of the patient to the care team—it is conceivable that CoCM improves these clinical experiences for all patients, but especially so for racially marginalized patients, who are typically treated with a more stark lack of advocacy and respect in traditional healthcare settings (12, 13, 35). Alternatively, it is possible that the often-observed racial disparities in healthcare settings are replicated in CoCM settings (54), and that White patients still benefit more from the model than do marginalized patients with regards to treatment perceptions. The present research was designed to examine these possibilities among a large cohort of individuals with co-occurring opioid use and mental health disorders.

## Current research

The present research had two aims. First, we examined whether exposure to CoCM, compared to enhanced usual care (EUC), was associated with improved perceptions of clinician communication, treatment timeliness, and overall treatment quality, among primary care patients presenting with opioid use disorder (OUD) co-occurring with depression and/or PTSD. Second, we examined whether these effects were moderated by race and ethnicity—specifically, if CoCM was associated with improvements in patients' perceptions of their treatment, was this relationship equally strong for patients belonging to advantaged and marginalized racial and ethnic groups? We tested these questions among primary care patients in rural and urban health clinics in New Mexico and California.

## Methods

### Procedures

Data for this study were part of a larger randomized control trial: Collaboration Leading to Addiction Treatment and Recovery from Other Stressors (CLARO). CLARO tested whether CoCM was more effective than enhanced usual care (EUC) for primary care patients with OUD co-occurring with depression and/or PTSD (55). Patients 18 years of age or older with OUD co-occurring with depression and/or PTSD were recruited for the trial from 18 rural and urban primary care clinics in New Mexico and California. These clinics belonged to four different health systems, which deliver care across one or multiple sites under shared administration. All clinics served low income and uninsured or underinsured individuals. The majority of patients were covered by Medicaid, Medicare or were self-pay on a sliding fee schedule. Sixteen out of 18 clinics were in areas with documented shortages of mental health care professionals. Clinics included a mix of community-based primary care facilities—such as federally qualified health centers providing integrated medical, behavioral health, and substance use services—and clinics affiliated with large academic health systems.

Upon enrollment, 797 participants completed a baseline interview, which included questions about demographics, social determinants of health, mental health, and substance use. Following the baseline assessment, participants were assigned to receive either CoCM or EUC based on a 1:1 randomization protocol. A stratified randomization design was used, with the strata determined by site and prior buprenorphine exposure, and included randomly permuted block sizes of 2 and 4. EUC group participants received usual primary care, which could include access to a non-study-trained community health worker. We offered training in the management of co-occurring disorders to primary care practitioners. Practitioners could apply training information to participants in both treatment groups. At the end of the six-month trial period, participants completed a follow-up interview, which included questions regarding physical and mental health symptoms, and subjective perceptions of care. Follow-up rates for overall treatment quality and clinician communication were 60% and 63%, respectively, with final analytic sample sizes of 482 and 503. Treatment Timeliness had fewer responses due to skip logic with a follow up rate of 42% and analytic sample size of 332.

Follow-up interviews were conducted verbally, primarily by telephone, and were documented in a web-based data capture system. The study was approved by RAND's institutional review board (IRB no. 2019-0509). Additional details about the study are available in the main outcomes paper (55).

### Measures

#### Outcomes

All outcome variables were self-reported by participants during research interviews. We specified three primary outcomes to model participants' subjective perceptions of care, using patient-level measures derived from the Consumer Assessment of Healthcare Providers and Systems (CAHPS) survey (56, 57). The CAHPS survey is known to have acceptable internal consistency and construct validity, and is considered an effective choice for assessing perceptions of care (58). Unlike typical satisfaction surveys, CAHPS focuses on objective behaviors (i.e., how often a provider communicated clearly). The first outcome represents quality of *clinician communication*, and was a composite derived using responses to six items, asking, in the past three months, how often did the people you went to for counseling or treatment: 1) listen carefully to you; 2) explain things in a way you could understand; 3) show respect for what you had to say; and 4) spend enough time with you. Item five asked, how often did you feel safe when you were with the people you went to for counseling or treatment; item six asked, were you involved as much as you wanted in your counseling or treatment.

The second outcome concerns the *treatment timeliness* component of the clinician relationship and captures participants' perception of getting treatment quickly. This outcome was a composite measure derived using responses to three items, asking, in the past three months: 1) how often did you get the professional counseling you needed on the phone or by video chat; 2) when you needed counseling or treatment right away, how often did you see someone as soon as you wanted; and 3) not counting times you needed counseling or treatment right away, how often did you get an appointment for counseling or treatment as soon as you wanted.

The third outcome measured *overall treatment quality*, and was based on one item that asked, what number would you use to rate all your counseling or treatment in the last 3 months. All items included “don't know” and “refuse” response options. Items within the clinician communication and treatment timeliness composites were measured using a 1 (never) to 4 (always) response scale. All participants were asked clinician communication questions, whereas the treatment timeliness items were only asked of participants who reported seeking mental health or substance abuse care. The overall treatment quality item was was asked of all participants and measured using a 0 (worst possible) to 10 (best possible) response scale. These values were later converted to a zero to one hundred scale (see data preparation).

#### Treatment group and key moderator of interest

Our primary independent variable was treatment group (CoCM vs. EUC), and our moderator was patient race and ethnicity, where we used self-reported race and ethnicity information to assign participants into one of three groups: Latine, non-Latine White, or another race/ethnicity. This third category included patients who identified as American Indian or Alaska Native, Asian, Native Hawaiian or Pacific Islander, Black or African American, and two or more races, but not Latine. We conducted our ethnic/racial groupings in this way due to sample size constraints in our patient sample, which was majority Latine. This grouping still enabled us to fulfill our main moderation investigation, which was to compare the perceptions of minoritized and advantaged racial and ethnic groups.

#### Control covariates

We included covariates in our model known or hypothesized to be associated with our primary outcomes, for which we sought to ensure balance to minimize potential confounds. These covariates included the Pain, Enjoyment and General Activity (PEG) scale to measure pain (59); the Alcohol Use Disorders Identification Test (AUDIT) (60); and the Patient-Reported Outcomes Measurement Information System (PROMIS) to measure OUD severity in the past 30 days (61). Mental and physical health functioning were captured respectively through the MCS and PCS of the 12-item Veterans RAND Health Survey (VR-12) (62), and a binary measure of suicidal ideation in the past 30 days was derived using the Columbia-Suicide Severity Rating Scale (C-SSRS) (63). Depression symptom severity was measured using the PHQ-9 (64), and PTSD symptom severity for participants with probable PTSD at baseline was measured using the PCL-5 (65). Participants' use of medication for opioid use disorder (MOUD), days of illicit opioid, any drug, and stimulant use (66), recent overdose, housing status, marital status, history of legal trouble and trauma, health system, education level, age, and sex on current birth certificate were collected through direct self-reports in the baseline interview.

### Data preparation

We followed a multi-step process to derive our outcome variables. Following standard CAHPS scoring protocol (56, 57), we mean-centered each outcome measure's component items and then implemented a case-mix adjustment (CMA) model on the mean-centered values, using patient age, education, physical health, mental health, and language of survey administration. Case-mix adjusted responses were converted to a zero to one hundred scale, and outcome variables were derived by taking the mean of the converted components. Lastly, we censored outcome values to a range of zero to one hundred, such that negative scores were converted to zeros, and scores greater than one hundred were converted to one hundred. We performed all data preparation in SAS 9.4 and ran the CMA model using the surveyreg procedure.

### Statistical analyses

For our main effect and moderator analyses, we modeled a linear relationship between each outcome and treatment assignment, adjusting for patient race/ethnicity and health system. In the main effects models, we assess for statistically significant differences at the 0.05 levels and highlighted our findings using figures that showcase adjusted means together with 95% confidence intervals for each CAHPS outcome. For the moderator analysis, we ran stratified models for each race/ethnicity group and assessed whether we had evidence of moderation by checking if the 95% confidence intervals overlapped. To account for non-response bias and imbalances on baseline covariates among responders at six-month follow-up, we calculated non-response and propensity score weights for each outcome using the control covariates described above as well as an indicator for treatment group when estimating non-response weights. We calculated non-response and propensity score weights using the twang package in R. We ran outcome models using ordinary least squares regression and included the weights in our final outcome models as survey weights with the survey package from Stata 17.

## Results

Table 1 presents the sociodemographic and clinical characteristics for each CAHPS outcome. The analytic sample for the treatment timeliness outcome (*n* = 332) had two statistically significant differences—those in the CoCM group were more likely to be on no MOUD at baseline compared to the EUC group (22.2% vs. 13%, *p* = 0.027) and had higher levels of pain (5.8 vs. 4.9, *p* = 0.003). The analytic sample for the clinician communication outcome (*n* = 503) had three statistically significant differences—those in the CoCM group were more likely to have current legal trouble compared to the EUC group (21.4% vs. 14.1%, *p* = 0.033) and had worse physical health (36.1 vs. 38.6, *p* = 0.04) and higher levels of pain (5.6 vs. 4.9, *p* = 0.008). The analytic sample for the overall treatment quality outcome (*n* = 482) had two statistically significant differences—those in the CoCM group were more likely to have current legal trouble compared to the EUC group (21.4% vs. 14.1%, *p* = 0.037) and had higher levels of pain (5.7 vs. 5.0, *p* = 0.009). We note that these differences disappear after applying the propensity score weight.

**Table 1:** Baseline Sociodemographic and Clinical Characteristics by Treatment Group.

Patient characteristics	Treatment timeliness		Clinician communication		Overall treatment quality	
	EUC	CoCM	EUC	CoCM	EUC	CoCM
	(*n* = 161)	(*n* = 171)	(*n* = 255)	(*n* = 248)	(*n* = 248)	(*n* = 234)
	*N* (%) or Mean (SD)	*N* (%) or Mean (SD)	*N* (%) or Mean (SD)	*N* (%) or Mean (SD)	*N* (%) or Mean (SD)	*N* (%) or Mean (SD)
Study *N*	161 (48.5)	171 (51.5)	255 (50.7)	248 (49.3)	248 (51.5)	234 (48.5)
Age in years, mean (sd)	41.2 (11.57)	40.8 (11.93)	41.0 (11.58)	41.2 (12.68)	41.1 (11.66)	41.1 (12.45)
Sex, as appears on current birth certificate, *N* (%)						
Male	64 (39.8)	68 (39.8)	107 (42.0)	103 (41.5)	105 (42.3)	95 (40.6)
Female	97 (60.2)	103 (60.2)	148 (58.0)	145 (58.5)	143 (57.7)	139 (59.4)
Race and Ethnicity, *N* (%)						
Non-Latine, White	46 (28.6)	37 (21.6)	64 (25.1)	62 (25.0)	62 (25.0)	55 (23.5)
Latine	103 (64.0)	117 (68.4)	167 (65.5)	166 (66.9)	163 (65.7)	159 (67.9)
Another race or ethnicity	12 (7.5)	17 (9.9)	24 (9.4)	20 (8.1)	23 (9.3)	20 (8.5)
Education, *N* (%)						
Less than high school	51 (31.7)	52 (30.4)	72 (28.2)	71 (28.6)	71 (28.6)	68 (29.1)
High school or equivalent	37 (23.0)	42 (24.6)	71 (27.8)	68 (27.4)	66 (26.6)	67 (28.6)
Some college or more	73 (45.3)	77 (45.0)	112 (43.9)	109 (44.0)	111 (44.8)	99 (42.3)
Marital status, *N* (%)						
Never married	59 (36.6)	57 (33.3)	93 (36.5)	84 (33.9)	91 (36.7)	79 (33.8)
Married/living with partner	64 (39.8)	61 (35.7)	98 (38.4)	90 (36.3)	96 (38.7)	86 (36.8)
Widowed/divorced/separated	38 (23.6)	53 (31.0)	64 (25.1)	74 (29.8)	61 (24.6)	69 (29.5)
Living in stable housing (3 months), *N* (%)						
No	19 (11.8)	19 (11.1)	26 (10.2)	24 (9.7)	27 (10.9)	24 (10.3)
Yes	142 (88.2)	152 (88.9)	229 (89.8)	224 (90.3)	221 (89.1)	210 (89.7)
Any current legal trouble, *N* (%)						
No	135 (83.9)	132 (77.2)	219 (85.9)	195 (78.6)*	213 (85.9)	184 (78.6)*
Yes	26 (16.1)	39 (22.8)	36 (14.1)	53 (21.4)	35 (14.1)	50 (21.4)
Days with opioid use (30 days), mean (sd)	6.2 (11.19)	6.2 (10.94)	6.4 (11.08)	6.3 (11.15)	6.3 (11.01)	6.2 (11.05)
PROMIS t-score (30 days)^a^, mean (sd)	51.8 (8.63)	53.2 (7.47)	52.4 (8.53)	52.4 (7.67)	52.4 (8.59)	52.4 (7.57)
Any opioid overdose events (3 months), *N* (%)						
No	154 (95.7)	163 (95.3)	248 (97.3)	237 (95.6)	241 (97.2)	223 (95.3)
Yes	7 (4.3)	8 (4.7)	7 (2.7)	11 (4.4)	7 (2.8)	11 (4.7)
Days with stimulant use (30 days), mean (sd)	5.2 (10.01)	4.0 (8.76)	4.9 (9.70)	3.8 (8.59)	5.0 (9.69)	3.7 (8.39)
Days with any drug use (30 days), mean (sd)	8.2 (12.07)	7.8 (11.71)	8.4 (12.05)	7.6 (11.72)	8.4 (12.02)	7.4 (11.52)
AUDIT sum (3 months)^b^, mean (sd)	3.7 (7.58)	4.1 (7.80)	3.7 (7.15)	4.0 (7.30)	3.7 (7.38)	4.0 (7.42)
MOUD history (30 days), *N* (%)						
No MOUD	21 (13.0)	38 (22.2)*	37 (14.5)	48 (19.4)	35 (14.1)	46 (19.7)
On methadone as prescribed	30 (18.6)	30 (17.5)	50 (19.6)	47 (19.0)	50 (20.2)	45 (19.2)
On buprenorphine as prescribed	102 (63.4)	86 (50.3)	156 (61.2)	133 (53.6)	152 (61.3)	123 (52.6)
On MOUD, never/sometimes prescribed	8 (5.0)	17 (9.9)	12 (4.7)	20 (8.1)	11 (4.4)	20 (8.5)
PHQ-9 score^c^, mean (sd)	14.1 (5.93)	13.9 (5.45)	13.7 (5.96)	13.4 (5.67)	13.8 (5.95)	13.4 (5.67)
Any history of trauma^d^, *N* (%)						
No trauma	11 (6.8)	9 (5.3)	15 (5.9)	22 (8.9)	15 (6.0)	19 (8.1)
Trauma, no interpersonal violence	78 (48.4)	100 (58.5)	135 (52.9)	142 (57.3)	129 (52.0)	134 (57.3)
Trauma with interpersonal violence	72 (44.7)	62 (36.3)	105 (41.2)	84 (33.9)	104 (41.9)	81 (34.6)
PCL-5 score^e^, mean (sd)	39.5 (16.26)	40.2 (17.93)	37.2 (16.66)	37.4 (18.17)	37.2 (16.67)	37.6 (18.18)
Suicidal ideation (30 days), *N* (%)						
No	109 (67.7)	118 (69.0)	176 (69.0)	180 (72.6)	173 (69.8)	169 (72.2)
Yes	52 (32.3)	53 (31.0)	79 (31.0)	68 (27.4)	75 (30.2)	65 (27.8)
VR-12 mental health component score^f^, mean (sd)	31.6 (12.47)	31.6 (12.30)	33.3 (12.60)	33.4 (12.89)	33.1 (12.62)	33.3 (12.88)
VR-12 physical health component score^f^, mean (sd)	38.6 (13.25)	36.3 (12.92)	38.6 (13.34)	36.1 (13.50)*	38.4 (13.38)	36.2 (13.32)
PEG score (avg)^g^, mean (sd)	4.9 (3.06)	5.8 (2.76)*	4.9 (3.06)	5.6 (2.81)*	5.0 (3.05)	5.7 (2.80)*
Health system, *N* (%))						
Health system 1	81 (50.3)	79 (46.2)	122 (47.8)	119 (48.0)	119 (48.0)	113 (48.3)
Health system 2	8 (5.0)	9 (5.3)	10 (3.9)	10 (4.0)	10 (4.0)	9 (3.8)
Health system 3	65 (40.4)	74 (43.3)	106 (41.6)	100 (40.3)	103 (41.5)	95 (40.6)
Health system 4	7 (4.3)	9 (5.3)	17 (6.7)	19 (7.7)	16 (6.5)	17 (7.3)

EUC, Enhanced Usual Care; CoCM, Collaborative Care Model; PROMIS, Patient-Reported Outcomes Measurement Information System; MOUD, medications for opioid use disorder; AUDIT, Alcohol Use Disorders Identification Test; PHQ-9, Patient Health Questionnaire-9; PCL-5, Post-Traumatic Stress Disorder Checklist for Diagnostic and Statistical Manual of Mental Disorders 5; VR-12, Veterans RAND 12 Item Health Survey; PEG, Pain, Enjoyment, General Activity tool.

a PROMIS t-score measures severity of opioid use disorder, with range of 0 (best) to 100 (worst), centered at 50 with a standard deviation of 10.

b AUDIT sum measures severity of alcohol use, with range of 0 (best) to 40 (worst).

c PHQ-9 measures severity of depression symptoms, with range of 0 (best) to 27 (worst).

d History of trauma is coded using the worst traumatic event reported as part of the PCL-5 assessment. Events are categorized as trauma with interpersonal violence if they include harm intentionally inflicted on the participant by another person.

e PCL-5 measures severity of PTSD symptoms, with range of 0 (best) to 80 (worst).

f Mental health and physical health component scores measured with VR-12, with range of 0 (worst) to 100 (best), centered at 50 with a standard deviation of 10.

g PEG (avg) measures severity of pain, with a range of 0 (best) to 10 (worst).

* *P* < 0.05.

Differences in sample size across measures are due to a combination of different question gates and missing data.

**Figure 1:**
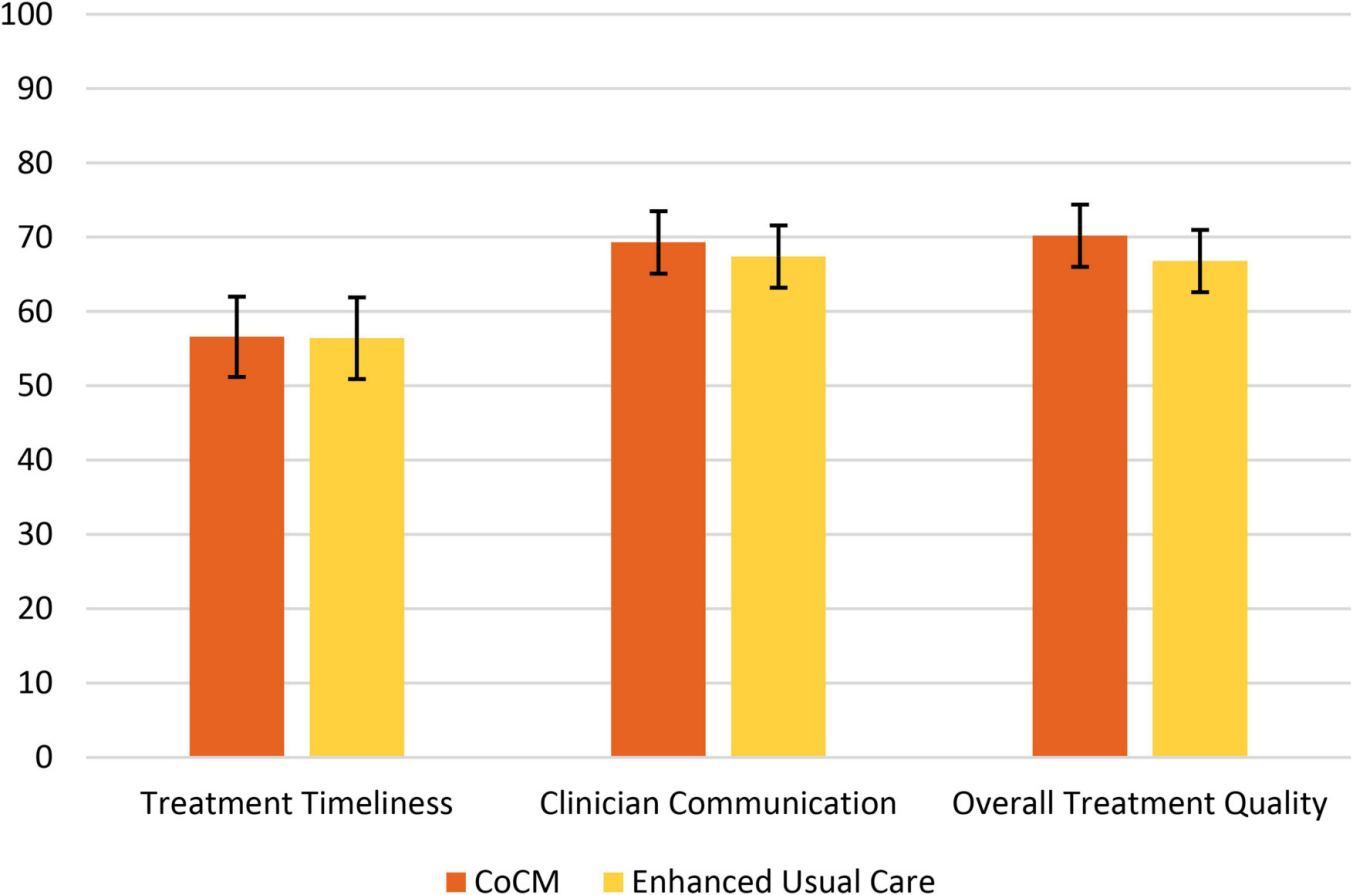
CAHPS Outcome Adjusted Means and 95% Confidence Intervals by Treatment Group.

Figure 1 shows the adjusted means and 95% confidence intervals for each CAHPS outcome by treatment group from the propensity score-weighted main effect analysis. We observed no significant main effect of treatment group (CoCM vs. EUC) on any of the CAHPS outcomes: treatment timeliness: 56.6 [51.2–62.0] vs. 56.4 [50.9–61.8], *p* = 0.947; clinician communication: 69.3 [65.1–73.7] vs. 67.4 [63.2–71.6], *p* = 0.540; overall treatment quality: 70.2 [66.0–74.4] vs. 66.8 [62.6–71.1], *p* = 0.268.

**Figure 2:**
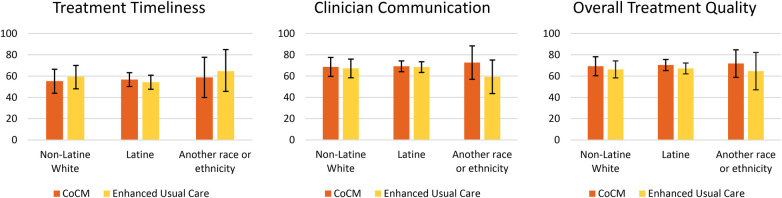
Ethnicity Stratified Analysis of CAHPS Outcome Adjusted Means by Treatment Group.

Figure 2 shows the adjusted means and 95% confidence intervals for each CAHPS outcome by treatment group from the stratified models, again adjusting for propensity score weights. We continue to observe no significant effect of treatment group (CoCM vs. EUC) on any of the CAHPS measures when stratified by each race/ethnicity group. In all cases, the 95% confidence intervals for the adjusted means of treatment groups were overlapping for each race/ethnicity group. For example, for treatment timeliness, the adjusted means for CoCM vs. EUC, respectively, were: 56.7 [50.2–63.2] vs. 54.2 [47.5–60.9], *p* = 0.598 for Latine, 55.2 [43.9–66.5] vs. 59.4 [48.8–69.9], *p* = .598 for Non-Latine White, and 58.8 [39.9–77.8] vs. 64.6 [44.3–84.8], *p* = .671 for another race/ethnicity.

## Discussion

The current study examined whether randomization to CoCM is associated with improved perceptions of treatment among patients with co-occurring disorders, and whether this effect is moderated by race and ethnicity. Surprisingly, we identified no significant differences in perceptions of clinician communication, treatment timeliness, or overall treatment quality between collaborative care and usual care conditions, regardless of patient race and ethnicity.

Our results contrast with prior literature suggesting that CoCM is associated with greater overall patient satisfaction (3, 8, 14), as well as improved perceptions of clinician communication (3, 21) and treatment timeliness (25). This prior literature is embedded within a broader literature suggesting that changes in clinical care processes—as is the case in a CoCM intervention—can lead to improvements in patient perceptions of care (67). Our results did not bear this out: we observed no evidence of a difference between CoCM and EUC conditions on our three metrics.

It is possible that the divergence between the present results and those in other studies stems from a difference in patient population. The studies that have considered the impact of CoCM on patient perceptions of treatment have largely done so among populations with mental health disorders such as depression (3, 8, 14, 21). The present study recruited patients with OUD co-occurring with depression and/or PTSD—a population that is known to be complex to treat, given the enhanced stigma, symptom severity, and barriers to effective treatment that often coincide with these co-occurring disorders (68, 69). Given that CoCM typically involves improved integration between providers and includes a care manager who advocates for the patient within the care team, it is plausible that CoCM would be particularly effective at improving perceptions of care among patients with complex and stigmatized conditions. These patients might often face the challenge of navigating multiple disconnected providers in traditional healthcare settings, and as such, CoCM might be an especially welcome change. However, given that patients with more complex or stigmatized conditions often enter care with greater need, improvements that may feel meaningful to other patient populations could be perceived as insufficient among these groups. Prior research suggests that patients often evaluate care relative to preconceived “ideals” rather than their actual experiences (15), and these ideals may be especially elevated among those with greater need. Moreover, literature on patient experience indicates that prior interactions with the healthcare system can shape perceptions of new encounters (15). If patients with more complex disorders have more extensive prior healthcare involvement, they may be less sensitive to the improvements of a single six-month intervention. Future research should further examine how patients with co-occurring disorders perceive clinician communication, treatment timeliness, and overall care quality to better understand the factors influencing their treatment experiences.

It is also possible that our results reflect spillover effects. Because providers cared for participants in both the treatment and control arms, it is possible that all patients, across conditions, were receiving some of the benefits of CoCM, which may have dampened our ability to detect an effect of the intervention on participants' overall perceptions of treatment (55). Comparing our results to benchmark scores from other datasets proved challenging due to the unique characteristics of our participant population and our specific analysis procedures. We were therefore unable to determine with confidence whether perceived treatment quality in our sample were relatively high or low compared to other studies.

In the present study, the association between assignment to CoCM and perceptions of clinician communication, treatment timeliness, and overall treatment quality did not significantly differ between racial and ethnic groups. Again, this pattern diverges from prior literature regarding the differential impact of CoCM on clinical outcomes for members of different racial and ethnic groups (12, 13, 54). Some literature has determined that in CoCM settings, racial minority patients demonstrate greater progression in mental health recovery than White patients (13). Other studies have found that compared to usual care, where White patients often demonstrate greater recovery progress, under CoCM, racial disparities in recovery dissipate (12). Of course, these studies each considered clinical mental health outcomes (severity of depression and anxiety) as their outcome, rather than subjective perceptions of treatment, as in the present study. The small body of research that has considered the subjective treatment perceptions of minoritized patients largely found CoCM to improve treatment satisfaction, though the effect on clinician relationships was not specifically examined, nor were these effects explicitly compared to those among racially advantaged patients, as in the present study (52, 53).

Furthermore, the patient population in the present study includes individuals with co-occurring OUD and mental health disorders, which involves additional complexity compared to the populations in prior literature (12, 13, 52, 53). To our knowledge, the present work is the first to examine racial and ethnic patterns in subjective perceptions of treatment in response to CoCM—specifically regarding clinician communication, treatment timeliness, and overall treatment quality—within this stigmatized population (68). While the association between assignment to CoCM and perceptions of clinician communication, treatment timeliness, and overall treatment quality did not significantly differ between racial and ethnic groups, we cannot interpret null results as confirmed *absence* of effects. Additional research—with attention to the specifics of patient population and potential intersecting stigma—will be necessary to further explore these patterns.

While our results conflict with prior research (12, 13, 54), they may be reflective of the conclusions of a smaller body of studies which argue that particular localized contexts may reveal unique patterns regarding racial and ethnic differences, or lack thereof, in health care experience (70–72). These studies have highlighted that, while racial and ethnic disparities in health care outcomes and experiences undoubtedly persist across the U.S., the level of analysis at which they are examined can influence findings (70) and in some contexts, they may not emerge (70, 72). CLARO was conducted in health systems across New Mexico and California, both of which have developed comprehensive health equity plans in the past several decades (73–75). Beginning in 2005, New Mexico conducted a thorough reform of their mental health services to provide more culturally competent care to their majority-Latine population, including non-English language services and increased cultural sensitivity training for clinicians (74, 75). Similarly, since 2016, the California Department of Public Health has been working in collaboration with community-based partners to implement a State Health Improvement Plan, specifically designed to advance health equity and improve community health for “populations experiencing significant disparities across health outcomes” (73). Given this context, it is possible that within New Mexico and California specifically, clinician relationships and treatment experiences across racial and ethnic groups are already more thoroughly supported compared to other locales, and as such, we did not observe further racial and ethnic differences in response to CoCM.

It is also possible that our inability to detect a moderation by race and ethnicity was due to measurement or research practice constraints (76, 77). For instance, it is possible that our measures of perceived clinician communication, treatment timeliness, and overall treatment quality, did not capture meaningful dimensions of these constructs for non-White patients (76). Research has suggested that terms like “respect”—a core component of the clinician communication questions—may carry different meanings for White patients than they do for Black and Latine patients, and that common definitions of respect often do not encapsulate the salient meaning for Black and Latine patients (76). For this reason, it is possible that our measures did not capture meaningful dimensions of respect or disrespect for racially and ethnically marginalized patients. Relatedly, recent work suggests that when examining the perceptions of racially minoritized individuals, utilizing culturally-relevant language and materials—ideally delivered by research team members who have developed a relationship of trust with the patient—are the most effective at fully capturing nuanced experience (77). Future work might endeavor to more comprehensively capture racial trends in perceived quality of care through these means.

Our results are specific to the patient population and socio-geographical landscape in which the study took place. We cannot generalize our findings to all CoCM interventions, nor even all CoCM interventions involving depression, PTSD, or OUD. The co-occurring nature of the disorders in our patient population, and the specific social construction of New Mexico and California health systems, each offer important context that limit the generalizability of our findings. Future research in other patient populations and other social and geographical settings will be necessary to demonstrate the ubiquity of our findings.

This study had several limitations. While we compared treatment perceptions from patients *assigned* to CoCM vs. usual care conditions, we did not examine true *receipt* of collaborative care—namely, contact with the care manager. It is possible that our results were diluted by individuals in the intervention condition who did not fully experience the intervention, although most patients did have contact with the care manager (78). Additionally, follow-up rates for our outcome measures were relatively low, ranging from 42% to 63%. While we accounted for potential bias by applying non-response weights for each outcome, it is possible that the substantial non-response rates in the present data may have introduced bias for which we were unable to account.

Furthermore, we did not consider moderators beyond race and ethnicity of the relationship between treatment condition and subjective perceptions of care. Prior literature suggests that factors such as patient-level attachment or relationship style can moderate the relationship between CoCM assignment and treatment satisfaction (79). As such, it is possible that other intersecting, undetected covariates may have influenced our outcomes. Furthermore, our categorization of racial and ethnic groups inevitably involved collapsing potentially meaningful identity-based differences between individuals (80), which may have contributed to within-group variance. Future research might consider modes of categorization that more meaningfully capture racial and ethnic groupings. Additionally, given the relatively small number of participants from some racial and ethnic groups, our analyses may have been underpowered to detect meaningful differences across these subgroups when examining moderation. Future studies with larger and more diverse samples will be important to more definitively assess potential differences in outcomes by race and ethnicity. Finally, it is possible that a longer intervention period, with follow-up more than six months later, would have been required to detect meaningful shifts in participants' perceptions of care.

The present work examined the effects of collaborative care (CoCM), relative to enhanced usual care, on patient perceptions of clinician communication, treatment timeliness, and overall treatment quality among primary care patients with OUD co-occurring with depression and/or PTSD in New Mexico and California. Additionally, the study explored whether these relationships were influenced by patient race or ethnicity. Diverging from patterns in prior research, we found no evidence for an effect of CoCM relative to EUC on patients' perceptions of clinician communication, treatment timeliness, or overall treatment quality, and no evidence of moderation by patient race or ethnicity. Together, our findings highlight the need to consider localized context and specific research practices when examining the effects of clinical interventions on perceptions of care among stigmatized patient populations.

## Data Availability

The raw data supporting the conclusions of this article will be made available by the authors, without undue reservation.
